# Butyrate and Propionate Restore the Cytokine and House Dust Mite Compromised Barrier Function of Human Bronchial Airway Epithelial Cells

**DOI:** 10.3390/ijms22010065

**Published:** 2020-12-23

**Authors:** Levi B. Richards, Meng Li, Gert Folkerts, Paul A.J. Henricks, Johan Garssen, Betty C.A.M. van Esch

**Affiliations:** 1 Division of Pharmacology, Utrecht Institute of Pharmaceutical Sciences, Faculty of Science, Utrecht University, 3584 CG Utrecht, The Netherlands; l.b.richards@amsterdamumc.nl (L.B.R.); m.liuu@outlook.com (M.L.); g.folkerts@uu.nl (G.F.); P.A.J.Henricks@uu.nl (P.A.J.H.); j.garssen@uu.nl (J.G.); 2 Department of Respiratory Medicine, Amsterdam University Medical Centres (AUMC), University of Amsterdam, 1105 AZ Amsterdam, The Netherlands; 3 Nutricia Research, 3584 CT Utrecht, The Netherlands

**Keywords:** short-chain fatty acids, airway epithelial barrier function, ZO-1, MAPK signalling pathways

## Abstract

Barrier dysfunction of airway epithelium contributes to the development of allergies, airway hyper-responsiveness and immunological respiratory diseases. Short-chain fatty acids (SCFA) enhance and restore the barrier function of the intestinal epithelium. This study investigated whether acetate, propionate and butyrate enhance the integrity of bronchial epithelial cells. Differentiating human bronchial epithelial cells (16HBE) grown on transwells were exposed to butyrate, propionate and acetate while trans-epithelial electrical resistance was monitored over time. Restorative effects of SCFA were investigated by subsequent incubation of cells with IL-4, IL-13 or house dust mite extract and SCFA. SCFA effects on IL-4-induced cytokine production and the expression of zonula occludens-1 (ZO-1) and Mitogen-activated protein kinases (MAPK) signalling pathways were investigated by ELISA and Western blot assays. Propionate and butyrate enhanced the barrier function of differentiating 16HBE cells and induced complete recovery of the barrier function after exposure to the above-mentioned stimuli. Butyrate decreased IL-4-induced IL-6 production. IL-4 decreased ZO-1 protein expression and induced phosphorylation of extracellular signal-regulated protein kinases 1/2 (ERK1/2) and c-Jun N-terminal kinases (JNK) in 16HBE cells, both of which could be restored by SCFA. SCFA showed prophylactic and restorative effects on airway epithelial barrier function, which might be induced by increased ZO-1 expression.

## 1. Introduction

Asthma is a common chronic disease that poses a major burden on the world’s communities. Currently, no therapy is available to cure asthma, and therapeutics are aimed at reducing symptoms [1]. Current clinical therapies based on inhaled corticosteroids and long acting ß_2_ receptor agonists are effective in controlling asthma in most, but not all, patients. Eosinophilic asthma is an inflammatory disease of the airways characterised by infiltration of eosinophils, basophils, mast cells and CD4+ T helper (Th) cells into the airway submucosa. Th2 cells and type 2 innate lymphoid cells (ILC2s) producing IL-4, IL-5 and IL-13 are now recognised as important cells underlying eosinophilic asthma [2]. Epithelial cells are the first innate immune cells to interact with antigens and pathogens, and the airway epithelium plays an essential role in the protection responses to allergens, pathogens and environmental pollutants that contribute to asthma pathogenesis [3,4,5]. The epithelial barrier is constituted by the formation of complexes that consist of tight junction proteins together with adherens junction proteins and desmosomes [5,6,7,8]. The complexes form intercellular contacts between the epithelial cells, thereby limiting the passage of liquids and macromolecules across the epithelium via paracellular diffusion [9,10]. Under physiological conditions, the integrity of the epithelium is preserved by the tight junction proteins, such as zonula occludens-1 (ZO-1) [10,11].

Dysfunction of the tight junctions has been identified in asthma patients, possibly indicating a role for the airway epithelium in the pathophysiology of asthma [12]. Disruption of tight junction proteins is regarded as one of the earliest hallmarks of epithelial injury, which subsequently leads to the loss of cell polarity and decreases trans-epithelial electric resistance (TEER) in cultured airway epithelial cells. Damage to the epithelial barrier enables infiltration of the submucosa of the airways by allergens and induces activation of immune cells. Therefore, disruption of the regulatory function of the airway epithelial barrier could be an aggravating and possibly initiating factor of the chronic airway inflammation observed in asthma [3,4,5].

Triggers of asthma initiate the inflammatory response and the release of epithelium-derived cytokines. These epithelium-derived cytokines are responsible for the local production of IL-4, IL-5 and IL-13 by ILC2 and Th2 cells (Type 2 immune cells), which contribute to airway hyper-responsiveness and inflammation of the lungs [2]. The functionality of the airway epithelium can significantly decrease when epithelial cells are exposed to allergens, pathogens or pollutants. House dust mite (HDM) allergens, one of the most important environmental triggers of allergic asthma, and the Type 2 cytokines IL-4 and IL-13 can cause barrier dysfunction of the airway epithelium [7,9,13]. Analysis of primary airway epithelial cells obtained from asthmatic subjects revealed a diminished epithelial barrier function associated with decreased ZO-1 expression [12,14]. The expression of ZO-1 can be modulated by Mitogen-activated protein kinases (MAPK) signalling pathways leading to altered permeability, and most studies demonstrated the relationship between extracellular signal-regulated protein kinases ½ (ERK1/2) and ZO-1 [15]. Improving or restoring compromised airway epithelial barrier function is an interesting therapeutic strategy for the treatment of allergic diseases.

The bacterial fermentation of different dietary fibres leads to an increase in concentrations of several short-chain fatty acids (SCFA), especially butyrate, propionate and acetate, in the colon [16,17,18]. SCFA can be absorbed by the colonocytes, after which they are used as an energy source for cellular metabolism. Fatty acids that manage to escape the metabolism of the colonocytes can diffuse through the basolateral membrane and reach the hepatic portal vein. The beneficial effects of SCFA on inflammatory diseases of the intestinal tract have been known for some time. Many in vitro studies have been conducted into the effects of SCFA in the intestinal epithelium and demonstrated that SCFA, especially butyrate, are able to reinforce the barrier function of the intestinal epithelium [19,20,21,22,23,24,25]. However, data on the modulating effects of SCFA in lung diseases and particularly the effect on airway epithelium are limited [26].

Therefore, the aim of the present in vitro study was to determine whether SCFA can strengthen the barrier function (TEER) of airway epithelial cells and whether SCFA can be used for the treatment of existing damage to the barrier caused by exposure to the environmental allergen house dust mite and Type 2 cytokines IL-4 and IL-13. Furthermore, we investigated whether SCFA affected the expression of the tight junction protein ZO-1 and the MAPK signalling pathways.

## 2. Results

### 2.1. Assessment of Cell Damage

No indication of cytotoxicity could be observed following 72 h incubation of epithelial cells with SCFA and HDM at concentrations applied in the following experiments. Except for the positive control (1% Triton X-100 solution in culture medium), no significant differences in cytotoxicity were seen between applied treatments and the control. Similarly, no significant differences in cytotoxicity were found between the applied concentrations of SCFA and the control after daily administration of the fatty acids for 11 days.

### 2.2. The Effect of SCFA on the Development of the Barrier Integrity of 16HBE Cells

**Figure 1:**
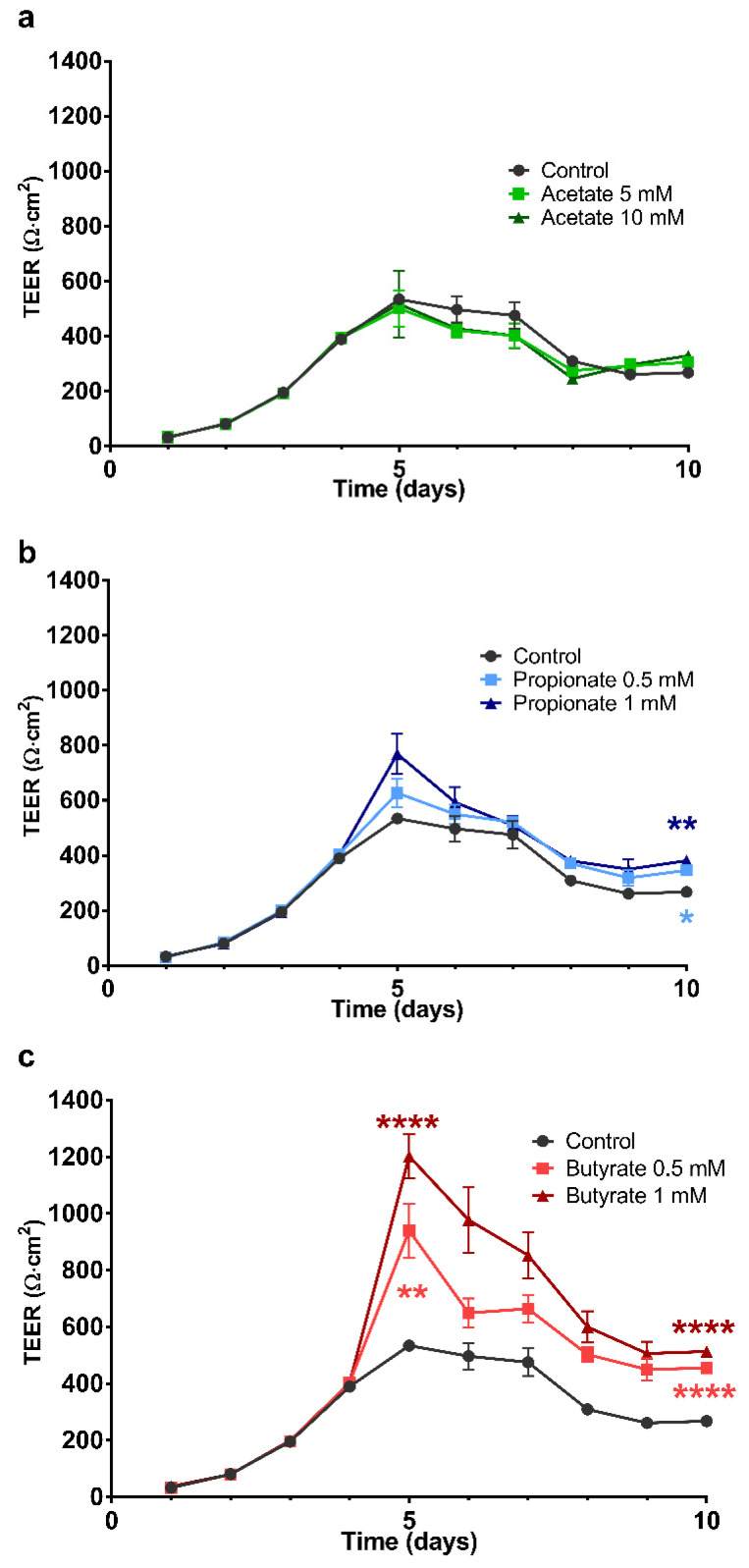
The effects of short-chain fatty acids (SCFA) on the development of the barrier function of 16HBE grown on transwells expressed as a function of time. Barrier function was determined daily before replacement of the medium using trans-epithelial electric resistance (TEER) measurements and were corrected for the background resistance due to the membrane and medium, and membrane surface. Starting from day 4 after seeding, the medium in the basolateral compartment of the transwells was replaced daily by a medium containing different concentrations of SCFA. (**a**–**c**) show the effects of SCFA on the barrier function when treatment was continued until day 10. The symbols shown correspond to the statistical tests performed on the data from measurements on days 5 and 10. All data are expressed as mean ± SEM of *n* = 3 independently performed experiments. **: *p* < 0.01 and ****: *p* < 0.0001 when compared to the control group.

The effects of repeated daily administration of SCFA on the development of the barrier formation of 16HBE cells were investigated. Figure 1 illustrates the development of TEER as a function of time, in which Figure 1a (acetate), b (propionate) and c (butyrate) show the effects of repeated administration for 6 days starting on day 4 when the cells were grown to confluency and ending on day 10. On day 5 and 10, the TEER values in controls were 534 ± 6 and 268 ± 14 Ω·cm^2^, respectively. Butyrate concentration-dependently increased the TEER values at day 5 to 940 ± 95 Ω·cm^2^ and 1203 ± 77 Ω·cm^2^ corresponding with 0.5 and 1 mM butyrate, respectively. The observed significant increases in TEER persisted to day 10 (after 6 days of treatment). Furthermore, significant results were obtained with propionate starting on day 10 (TEER 347 ± 14 and 382 ± 15 Ω·cm^2^ corresponding to 0.5 and 1 mM propionate, respectively). There were no significant differences between the incubations with acetate and the control.

### 2.3. Butyrate and Propionate Restored the Barrier in IL-4 Compromised 16HBE Cells

**Figure 2:**
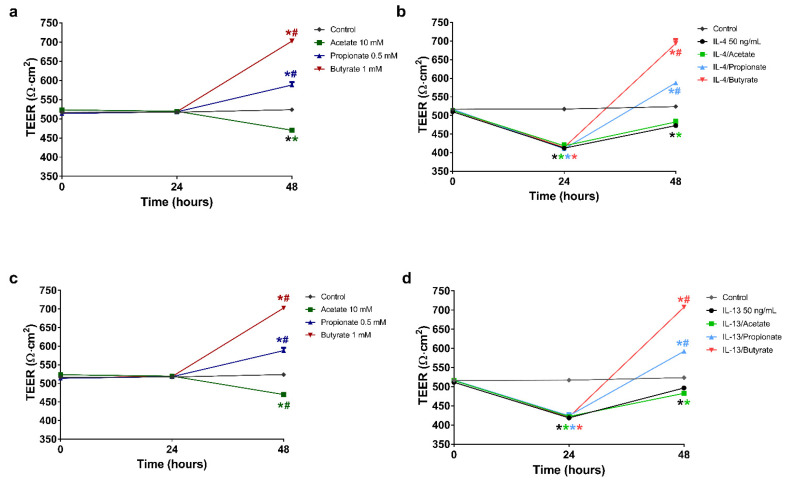
The effects of SCFA on the recovery of the barrier function of airway epithelium after stimulation with IL-4 or IL-13. 16HBE cells were grown on transwells for 14 days with daily refreshment of the culture medium. The cells were then stimulated at the basolateral side of the membrane with 50 ng/mL IL-4 or IL-13 for 24 h. After stimulation, the basolateral medium was replaced with medium alone or medium containing 10 mM acetate, 0.5 mM propionate or 1 mM butyrate. The barrier function was determined via TEER measurements before stimulation, before the addition of SCFA and 24 h after replacement of the basolateral medium. The values measured were corrected for background resistance and membrane surface. (**a**,**c**) show the effects of 10 mM acetate, 0.5 mM propionate and 1 mM butyrate on the barrier without stimulation. (**b**,**d**) show the effects of these SCFA on the restoration of the barrier function after stimulation with IL-4 or IL-13, respectively. All data are expressed as mean ± SEM of *n* = 3 independently performed experiments. *: *p* < 0.0001 when compared to control, #: *p* < 0.0001 when compared to IL-4 or IL-13. Coloured versions of these symbols indicate significant differences in the aforementioned comparisons for the treatment groups corresponding to the colour shown in the figure legends.

Pre-incubation with IL-4 (50 ng/mL) for 24 h, resulted in a significant reduction in TEER compared to control (Figure 2a,b; 413 ± 2 and 517 ± 2 Ω·cm^2^, respectively). Replacement of the basolateral medium with fresh culture medium without IL-4 resulted in a partial recovery of the TEER value during the next 24 h. Nevertheless, the TEER value remained significantly lower when compared to the control group (473 ± 5 and 524 ± 3 Ω·cm^2^, respectively). Similar results were observed after incubation with acetate (10 mM) following IL-4 exposure, which resulted in a significantly lower TEER value relative to the control group (470 ± 4 and 524 ± 3 Ω·cm^2^, respectively). In contrast, propionate (0.5 mM) or butyrate (1 mM) incubation without prior IL-4 stimulation resulted in significantly higher TEER values when compared to the control and the IL-4 stimulated group (588 ± 7, 703 ± 4, 524 ± 3 and 473 ± 5 Ω·cm^2^, respectively). Interestingly, both propionate and butyrate incubation following IL-4 exposure reversed the decrease in TEER values induced by IL-4, into an increase up to the levels of propionate and butyrate alone and far above control and the IL-4 stimulated group at 48 h (588 ± 1, 693 ± 11, 524 ± 3 and 473 ± 5 Ω·cm^2^, respectively).

### 2.4. Butyrate and Propionate Restored the Barrier in IL-13 Compromised 16HBE Cells

Pre-incubation with IL-13 (50 ng/mL) for 24 h induced a significant reduction in TEER when compared to control (Figure 2c,d; 420 ± 2 and 517 ± 2 Ω·cm^2^, respectively). Replacement of the basolateral medium with fresh culture medium without IL-13 caused a partial recovery of the TEER value during the next 24 h, although remaining lower compared to the control group (497 ± 2 and 524 ± 2 Ω·cm^2^, respectively). As observed with IL-4, acetate incubation following IL-13 exposure did not affect the increase in TEER values due to the replacement with fresh medium (without IL-13). Both groups, IL-13 and IL-13/acetate, remained below the control group. Again, as observed for IL-4, propionate and butyrate reversed the decrease in TEER values induced by IL-13, into an increase up to the levels of propionate and butyrate alone and far above control and the IL-13 stimulated group at 48 h (593 ± 2, 708 ± 2, 524 ± 3 and 497 ± 2 Ω·cm^2^, respectively).

### 2.5. Butyrate and Propionate Restored the Barrier in HDM Compromised 16HBE Cells

**Figure 3:**
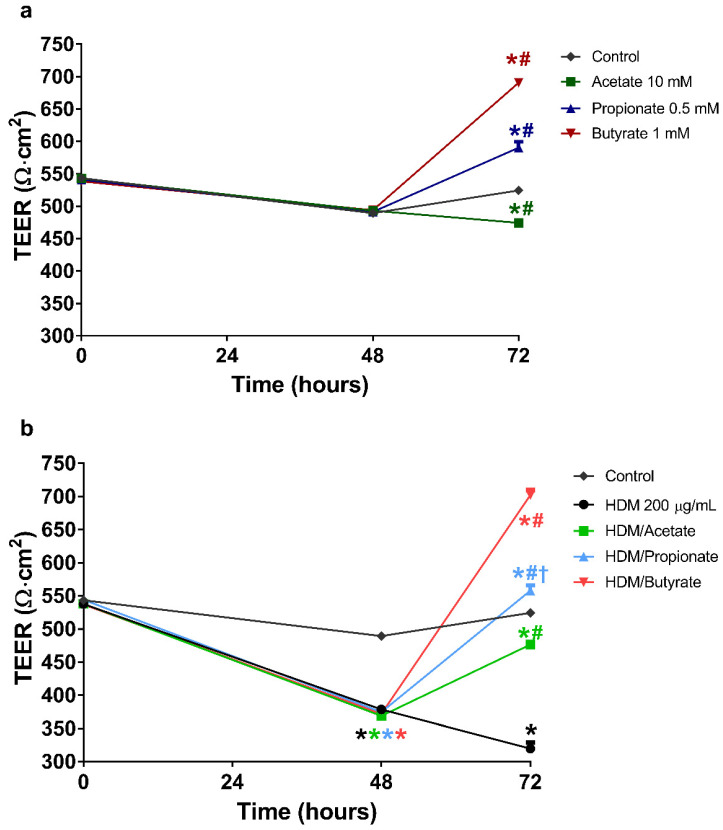
The effects of SCFA on the recovery of the barrier function of airway epithelium after stimulation with house dust mite extract (HDM). 16HBE cells were grown on transwells for 14 days with daily refreshment of the culture medium. The cells were then stimulated at the apical side of the membrane with 200 µg/mL HDM for 48 h. After the stimulation, the basolateral medium was replaced with medium alone or medium containing 10 mM acetate, 0.5 mM propionate or 1 mM butyrate. The barrier function was determined by means of TEER measurements before stimulation, before the addition of SCFA, and 24 h after replacement of the basolateral medium. The values measured were corrected for the background resistance and the surface of the membrane. (**a**,**b**) show the effects of 10 mM acetate, 0.5 mM propionate and 1 mM butyrate on the barrier function without prior stimulation and stimulation with HDM, respectively. All data are expressed as mean ± SEM of *n* = 3 independently performed experiments. *: *p* < 0.0001 when compared to control, #: *p* < 0.0001 when compared to IL-4 or IL-13 and †: *p* < 0.0001 when compared to the treatment group that only received the corresponding SCFA. Coloured versions of these symbols indicate significant differences in the aforementioned comparisons for the treatment groups corresponding to the colour shown in the figure legends.

Pre-incubation with HDM for 48 h resulted in a significant reduction in TEER when compared to the control group (Figure 3a,b; 371 ± 2 and 489 ± 4 Ω·cm^2^, respectively). Replacement of the apical compartment with fresh medium without HDM for 24 h resulted in an even further reduction in the barrier function, which remained significantly lower than the control (320 ± 9 and 524 ± 3 Ω·cm^2^, respectively). Incubation with acetate following HDM stimulation significantly increased TEER values when compared to the HDM treatment group (320 ± 9 Ω·cm^2^), although levels remained significantly lower compared to the control groups (474 ± 3, 477 ± 6 and 524 ± 3 Ω·cm^2^, respectively). Both propionate and butyrate alone increased the TEER values above control values. Interestingly, incubation with propionate or butyrate following HDM exposure resulted in significantly higher TEER values when compared to the control group and HDM stimulated group (558 ± 8, 702 ± 8, 524 ± 3 and 320 ± 9 Ω·cm^2^, respectively), indicating that the sustained decline in TEER induced by HDM was reversed by propionate and butyrate to TEER values above control.

### 2.6. Butyrate Inhibited IL-6 Production by IL-4 Stimulated 16HBE Cells

**Figure 4:**
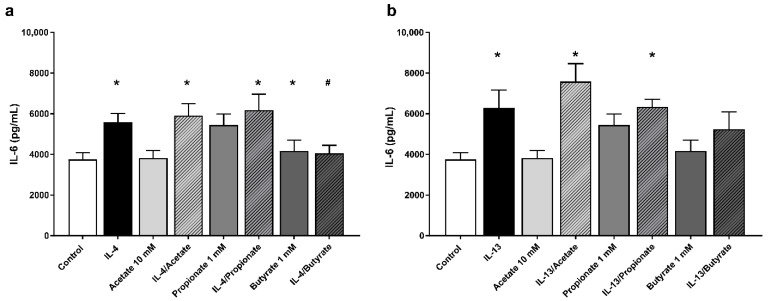
The effects of SCFA on the secretion of IL-6 by 16HBE cells after stimulation with T helper 2 (Th2)-associated pro-inflammatory cytokines. The effects of 10 mM acetate, 1 mM propionate or 1 mM butyrate incubation following stimulation with 50 ng/mL IL-4 and IL-13 are illustrated in (**a**) and (**b**), respectively. All data are expressed as mean ± SEM of *n* = 8 independently performed experiments. *: *p* < 0.05 when compared to the control group; #: *p* < 0.05 when compared to either IL-4 or IL-13 solely stimulated groups.

The effects of SCFA incubation on the secretion of IL-6 and IL-8 after stimulation with IL-4 or IL-13 were assessed using ELISA. In all experiments, stimulation with either IL-4 or IL-13 significantly increased secretion of IL-6 compared to the unstimulated control (Figure 4a,b; 5590 ± 429, 6291 ± 812 and 3750 ± 333 pg/mL, respectively). In contrast, no significant differences could be demonstrated in the secretion of IL-8 after stimulation with IL-4 or IL-13 compared to the unstimulated control. Incubation of SCFA without prior stimulation did not result in significant differences in the secretion of IL-6 or IL-8. Incubation with acetate or propionate following stimulation with either cytokine did not lead to significant differences in IL-6 and IL-8 secretion when compared to the solely stimulated groups. Incubation with butyrate led to a significant reduction in the secretion of IL-6 after stimulation with IL-4 compared to the solely stimulated group (4052 ± 392 and 5590 ± 429 pg/mL, respectively). However, no significant difference could be demonstrated in IL-6 secretion between the butyrate following IL-13 stimulation group and the exclusively stimulated group. Furthermore, butyrate incubation following stimulation with either cytokine did not result in significant differences in the secretion of IL-8 when compared to the solely stimulated groups.

### 2.7. SCFA Inhibited IL-4-Induced Activation of ERK1/2 and JNK Signalling Pathways

**Figure 5:**
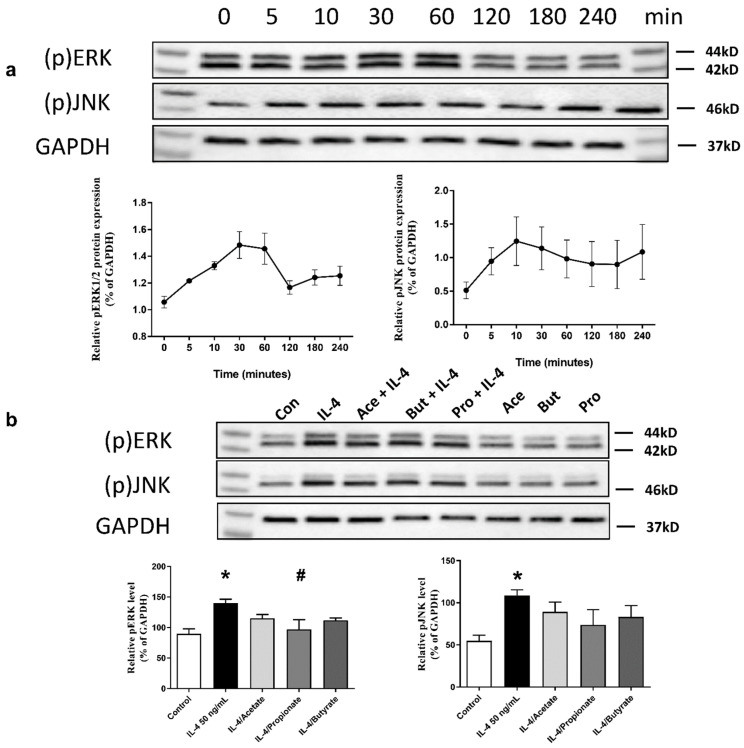
The effects of SCFA on IL-4-induced activation of Mitogen-activated protein kinases (MAPK) signalling pathways in 16HBE. (**a**) shows IL-4 (50 ng/mL) induced phosphorylation of extracellular signal-regulated protein kinases ½ (ERK1/2) and c-Jun N-terminal kinases (JNK) signalling pathways in 4 h. (**b**) shows the inhibitory effects of 10 mM acetate, 0.5 mM propionate and 1 mM butyrate on IL-4-induced activation of ERK1/2 and JNK signalling pathways. GAPDH is shown as a control. *n* = 3 independently performed experiments. *: *p* < 0.05 when compared to control, #: *p* < 0.05 when compared to the IL-4 solely stimulated group.

Confluent 16HBE were solely stimulated with IL-4 (50 ng/mL) for 4 h. Cell lysates were collected at 0, 5, 10, 30, 60, 120, 180 and 240 min. We found that IL-4-induced phosphorylation of ERK1/2 reached a peak after 30 min of stimulation, while phosphorylation of c-Jun N-terminal kinases (JNK) induced by IL-4 increased after 5 min of stimulation (Figure 5a). Confluent 16HBE were then stimulated with IL-4 (50 ng/mL) for 30 min in the presence or absence of acetate (10 mM), butyrate (1 mM) or propionate (0.5 mM). We found that propionate inhibited activation of ERK1/2 pathways. A trend towards inhibition of activation of ERK1/2 pathways was observed for butyrate (*p* = 0.051). No effect of SCFA was observed on JNK signalling pathways (Figure 5b).

### 2.8. SCFA Increased ZO-1 Expression in IL-4-Stimulated 16HBE

**Figure 6:**
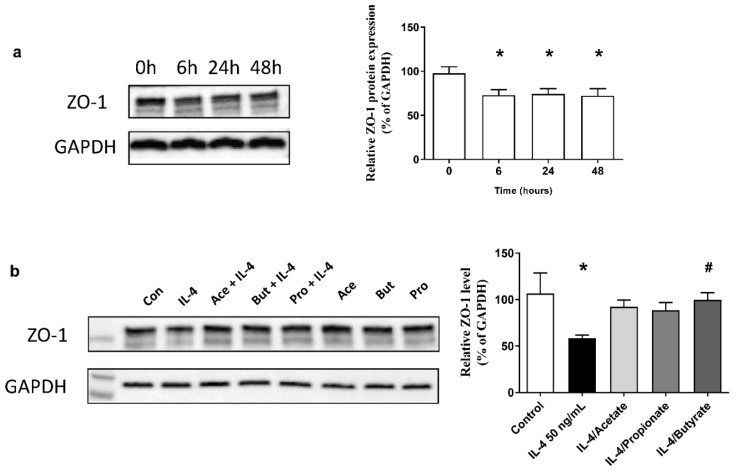
The effects of SCFA on IL-4-decreased zonula occludens-1 (ZO-1) expression in 16HBE. (**a**) shows that when confluent 16HBE were treated with or without IL-4 (50 ng/mL) for 6, 24 and 48 h and ZO-1 expression was decreased by IL-4 stimulation. (**b**) shows that treatment with 10 mM acetate, 0.5 mM propionate and 1 mM butyrate for 24 h restored ZO-1 expression in 24 h IL-4 stimulated 16HBE. GAPDH is shown as a control. *n* = 3 independently performed experiments. *: *p* < 0.05 when compared to control, #: *p* < 0.05 when compared to the IL-4 solely stimulated group.

To examine the effects of IL-4 on proteins associated with tight junctions, protein expression of ZO-1 was measured. Confluent 16HBE were stimulated with IL-4 (50 ng/mL) for 6, 24 and 48 h. We found that after stimulation with IL-4, ZO-1 expression was significantly decreased for up to 48 h (Figure 6a). Accordingly, the epithelial barrier function is also reduced. Confluent 16HBE were stimulated with IL-4 for 24 h, and cells were then treated with acetate (10 mM), propionate (0.5 mM) or butyrate (1 mM) for 24 h. Butyrate treatment restored ZO-1 expression in IL-4 stimulated epithelial cells (Figure 6b).

## 3. Discussion

The current study explored the effects of SCFA on the barrier function of human airway epithelial cells. We found that propionate and butyrate increased the barrier of the bronchial epithelium, as demonstrated by increased TEER values. Furthermore, SCFA induced complete recovery of the compromised airway epithelial barrier function caused by exposure of 16HBE cells to the environmental allergen HDM and the immune cell-related cytokines IL-4 and IL-13, which was associated with restored ZO-1 expression.

We demonstrated that incubation with butyrate dose-dependently enhanced the TEER of the epithelial cells on days 5 and 10. Furthermore, the effects of propionate treatment reached significance on day 10. Butyrate appeared to be more potent in enhancing TEER than propionate at similar concentrations. Moreover, as the effects of butyrate appeared to diminish on day 10, TEER values remained significantly increased in comparison to control. Recently it was shown that dietary intervention with SCFA ameliorated enhanced asthma susceptibility by modulating T-cells and dendritic cells [27]. Based on the current results, we can only speculate that the prophylactic use of SCFA could offer an opportunity in prevention strategies against the development of allergic diseases by increasing the barrier integrity of airway epithelium [26,27].

The airway epithelium represents an important barrier that protects against the intrusion of the internal environment by inhaled harmful substances from the external environment [5,6,7,8]. Patients with inflammatory respiratory diseases appear to be far more sensitive to exposure to allergens and infections, and it was observed that bronchial epithelial cells from patients with asthma proved to be much more susceptible to barrier dysfunction caused by exposure to HDM [4,12,14]. We observed that administration of HDM on the apical side of 16HBE cells, which simulates environmental exposure, compromised the barrier of 16HBE cells. In a recent paper, it was also shown that also other environmental factors, such as wild fire smoke extract, reduced the barrier function of airway epithelial cells [28].

Interaction of the epithelial cells with antigens can result in the secretion of mediators, which contribute to increased recruitment of immune cells into the airways [29,30,31,32,33]. Respiratory disorders, such as allergic asthma, are characterised by an influx of Th2 cells and eosinophils into the airways. The secretion of mediators by these immune cells, such as the allergy-related Type 2 cytokines IL-4 and IL-13, but also reactive oxygen species and proteases, contribute to the damage of the epithelium [2,34]. We applied IL-4 and IL-13 to the basolateral side of the 16HBE cells to investigate the effects of cytokines potentially produced by immune cells in the lungs. We observed that IL-4 and IL-13 compromised the barrier function of the airway epithelial cells, as shown by reduced TEER. Recently it was shown that IL-6/sIL6R induced loss of epithelial barrier integrity, which was related to a specific non Type 2 asthma phenotype [35,36]. We observed increased IL-6 production in stimulated 16HBE cells stimulated with IL-4. These data indicate that IL-6 produced by epithelial cells upon activation might further ameliorate the barrier function of airway epithelium.

The short-chain fatty acids acetate, propionate and butyrate are metabolites of the bacterial fermentation of indigestible carbohydrates and proteins in the colon [16,17,18]. The fatty acids possess potent anti-inflammatory effects and, therefore, can potentially be used for modulation of inflammatory disorders [20,37,38]. Many in vitro studies have been conducted into the effects of SCFA in the intestinal epithelium and demonstrated that SCFA, especially butyrate, are able to reinforce the barrier function of the intestinal epithelium [21,22,23,24,25]. Whether SCFA possess the capacity to restore the barrier and functionality of airway epithelial cells has not been investigated yet. Interestingly, both butyrate and propionate restored and even improved the barrier function of the compromised airway epithelium. This effect was present in 16HBE cells apically stimulated with HDM and the allergy-related cytokines IL-4 and IL-13, which were administered on the basolateral side of the epithelial cells. Acetate partially recovered barrier properties of 16HBE cells after stimulation with HDM, but no effects of acetate administration were observed after stimulation with IL-4 or IL-13. This dissimilarity in effect can possibly be attributed to the nature of the differences in stimuli applied in the experiments. Whereas cytokines are believed to exert barrier dysfunction via intracellular mechanisms, the serine proteases present in house dust mite are known to cleave tight junction proteins, including ZO-1, using its peptidase activity [39]. Butyrate also reduced the IL-4 induced epithelium-derived IL-6 secretion. In a recent paper, increased IL-6 production and reduced barrier function were associated with a reduction in the abundance of the tight junction proteins ZO-1 and Claudin-1 [28]. Nonetheless, contrary to our results, previous publications have reported pro-inflammatory effects of SCFA and poly-unsaturated fatty acids. However, these effects were observed in pulmonary cell types different from the cells investigated in this study and underlines the dependence of cell type on the nature of the inflammatory response elicited [40,41].

Tight junction proteins are the most apical intercellular junctions of epithelial cells. Under physiological conditions, tight junction proteins, such as ZO-1, cover the subapical regions of the epithelial cells and preserve the integrity of the epithelium. In asthma patients, a substantial decrease in the expression of ZO-1 can be observed [5,12,14]. The data in the current manuscript indicated that the breaking of the epithelial barrier was associated with decreased expression of ZO-1 in 16HBE cells. In agreement with the effects on TEER values, we observed that the decreased ZO-1 expression was restored by butyrate treatment. IL-4 decreased ZO-1 expression in airway epithelial cells is associated with the ERK1/2 signalling pathway [15,42]. Furthermore, it is reported SCFA can modulate the activation of protein kinase signalling pathways in intestinal epithelial cells [43]. Mitogen-activated protein kinases (MAPK), which includes ERK1/2, JNK and p38MAPK, are Ser/Thr protein kinases that respond to extracellular stimuli and regulate various cellular activities. We found that propionate treatment inhibited IL-4-induced activation of the ERK1/2 pathway, and a similar trend was observed for butyrate. In addition, these results suggest that the propionate, and possibly butyrate, induced effects on ZO-1 in airway epithelial cells are mediated by inhibiting the ERK1/2 signalling pathway. Moreover, in accordance with the observed effects on TEER after HDM exposure, acetate showed a trend towards recovery of the ZO-1 expression via ERK1/2 signalling pathway suppression.

Our results have to be viewed in light of a few limitations. First of all, due to the longitudinal nature of the experiments, the barrier function of the airway epithelial cells was assessed by measuring TEER. However, in future studies, more conclusive evidence of the effects SCFA on barrier function can be obtained by using destructive methods for specific time points via assays using paracellular transport markers, such as fluorescein-labelled dyes. Nonetheless, existing literature and our preliminary data demonstrate a strong association between TEER and permeability in 16HBE and other epithelial cells [44]. Second, the current study primarily focused on the effects of SCFA on barrier integrity. SCFA are known to be able to exert their effects via several mechanisms, namely via G-protein coupled receptor activation and/or histone deacetylases (HDAC) inhibition. Future studies need to be conducted to elucidate the mechanisms underlying our findings. Lastly, in the performed experiments, we used the immortalised human bronchial epithelial cell line 16HBE14o-. While it will be beneficial to replicate our findings using primary human bronchial cells, it needs to be appreciated that 16HBE14o- cells retain characteristic features of normal differentiated bronchial epithelial cells, including morphology, the ability to form tight junctions, ion transportation and cilia expression.

In the current study, experiments were performed by administering the SCFA at the basolateral side of the transwells, thereby mimicking exposure to SCFA originating from the systematic circulation. Although SCFA are known to be absorbed from the gastrointestinal tract, concentrations in the systemic circulation are generally low due to an extensive first-pass effect [18], thereby limiting the therapeutic applicability of oral administration as a potential route of administration. However, it is interesting to investigate whether SCFA can be applied as a therapeutic with a local route of administration to modulate the barrier function of airway epithelial cells. Furthermore, local routes of administration offer some advantages over systemic therapy, including a generally lower dose required to achieve therapeutic effects and reduced incidences of off-target effects [45,46,47]. In future studies, SCFA can be administered apically, thereby mimicking local exposure to these substances via inhalation.

Overall, we observed that butyrate showed the most potent effects on the reinforcement of the epithelial barrier function. Butyrate recovered the TEER in cytokine, and HDM compromised airway epithelial cells and increased ZO-1 expression in IL-4 compromised epithelial cells. Moreover, butyrate reduced the production of the inflammatory mediator IL-6 showing the capacity of butyrate to influence the production of pro-inflammatory mediators, which might contribute to a decreased integrity of the airway epithelium. Acetate showed some improvement in TEER in HDM compromised cells, but effects were small compared to the effects of butyrate and propionate.

A compromised epithelium is one of the symptoms of immunological respiratory diseases, including asthma, and is often responsible for increased sensitivity to inhaled substances and exacerbations of these airway disorders [10,24]. We showed, for the first time, that fermentation metabolites from the gut microbiome contribute to the recovery of barrier properties of airway epithelial cells, which may be mediated by increasing the expression of ZO-1.

## 4. Materials and Methods

### 4.1. Reagents

Minimal Essential Medium (MEM) and Dulbecco’s Modified Eagle Medium/F12 (DMEM/F12) with glutamax were purchased from Gibco (Thermo Fisher Scientific, Breda, Noord-Brabant, The Netherlands). Fetal bovine serum (FBS) was purchased from Bodinco (Alkmaar, Noord-Holland, The Netherlands). The bovine collagen type I solution was obtained from Advanced BioMatrix (CellSystems Biotechnologie Vertrieb, Troisdorf, Germany). The sodium salts of propionate and butyrate, as well as fibronectin from human plasma and penicillin–streptomycin (pen-strep) solutions, were purchased from Sigma (Breda, Noord-Brabant, The Netherlands). Sodium acetate was obtained from Merck Millipore (Amsterdam-Zuidoost, Noord-Holland, The Netherlands). Recombinant human IL-13 were purchased from R&D Systems (Abingdon, Oxfordshire, UK). Recombinant human IL-4 was purchased from ProSpec-Tany TechnoGene (Ness-Ziona, Israel). The HDM extract was obtained from GREER (Lenoir, NC, USA). The following monoclonal antibodies: anti-JNK1 + JNK2 (phospho T183 + Y185) antibody, anti-ERK1/2 (phospho Thr202/Tyr204) antibody, anti-GAPDH antibody, rabbit anti-mouse IgG H&L (HRP) conjugated antibody and goat anti-rabbit IgG H&L (HRP) conjugated antibody were purchased from Cell Signalling Technology (Leiden, Zuid-Holland, The Netherlands) and Abcam (Cambridge, UK).

### 4.2. Cells and Culture Conditions

The experiments were performed using the SV40-transformed and immortalised human bronchial epithelial cell 16HBE14o- (16HBE), which were kindly provided by the University of California, San Francisco, CA, USA [48,49]. The cells were grown in MEM supplemented with 10% (*V*/*V*) FBS in combination with 1% pen-strep. The plastic flasks were coated overnight with a coating solution consisting of fibronectin and collagen. The coating solution was prepared by diluting concentrated fibronectin derived from human plasma and bovine collagen type I solutions to a concentration of 30 µg/mL in DMEM/F12 with glutamax.

### 4.3. Trans-Epithelial Electrical Resistance (TEER)

16HBE cells were seeded at a density of 10^5^ cells per well on permeable transwell inserts with a polyester membrane with a diameter of 12 mm and a pore size of 0.4 µm (cat. no. 3460, Corning, Amsterdam, Noord-Holland, The Netherlands). Before seeding, the apical side of the insert was coated with a 30 µg/mL bovine collagen type I solution in 70% ethanol. The solution was filtered, after which the insert was coated by applying 70 µL to the apical side of the transwell. Then, the ethanol was allowed to evaporate overnight in a laminar flow hood under exposure to UV light to preserve sterility. The 16HBE cells were grown under liquid-covered culture conditions wherein the apical and basolateral fluid volumes were set at 250 and 900 µL, respectively. The epithelial barrier function of 16HBE was evaluated by measuring the trans-epithelial electrical resistance (TEER) using an EVOM volt–ohmmeter and an STX2 “chopstick” electrode (World Precision Instruments, Friedberg, Germany). The TEER values measured were corrected for the background resistance of an empty insert containing only medium and calculated as Ω·cm^2^.

### 4.4. The Effect of SCFA on the Development of the Barrier Integrity of 16HBE Cells

After seeding the cells to the transwells, the culture medium on both the apical and basolateral side was replaced daily with fresh culture medium. TEER values were determined daily before the replacement of the medium for 10 days, wherein the first measurement took place 24 h after seeding. Starting from day 4, when the cells were grown confluent, the cells were incubated with medium or medium containing 5 or 10 mM acetate, 0.5 or 1 mM propionate, 0.5 or 1 mM butyrate administered to the basolateral side of the inserts.

### 4.5. The Restorative Effects of SCFA after Barrier Disruption

In an additional set of experiments, 16HBE cells were cultured on inserts for 14 days with daily replacements of culture medium. Subsequently, the cells were stimulated for 24 h by replacing the basolateral medium with IL-4 or IL-13 (50 ng/mL) containing medium. Stimulation with house dust mite was accomplished by replacing the apical medium with a solution containing HDM (200 µg/mL) and incubated for 48 h. After the pre-incubation period, the basolateral medium was replaced with solutions of 10 mM acetate, 0.5 mM propionate or 1 mM butyrate in the medium. The cells were then incubated for an additional 24 h. TEER measurements were performed before the incubation with SCFA and 24 h after the addition of the last-mentioned substance. All solutions were warmed to 37 °C before they were added to the inserts.

### 4.6. Measurement of IL-6 and IL-8 Cytokine Levels

To investigate the effects of SCFA on the secretion of cytokines associated with asthma, ELISA measurements were performed. 16HBE cells were seeded at a density of 10^5^ cells per well on 24-wells plates and cultured until confluence was reached. Next, the cells were stimulated with either 50 ng/mL of recombinant IL-4 or IL-13 in the medium for 24 h. After stimulation, the cells were incubated for 24 h with medium or medium containing 10 mM acetate, 1 mM propionate or 1 mM butyrate. Subsequently, the supernatant was collected from the wells to assess the secretion of IL-6 and IL-8 using ELISA (Invitrogen, Thermo Fisher Scientific, Breda, Noord-Brabant, The Netherlands). The ELISAs were performed according to the manufacturer’s protocol. Supernatant cytokine levels were acquired by measuring absorbance using a 96-wells microplate reader and Microplate Manager 6 software (Bio-Rad, Hercules, CA, USA).

### 4.7. Western Blot

Confluent 16HBE were exposed to medium with or without IL-4 and HDM and then treated with SCFA. After treatment, cells were washed with cold PBS and lysed on ice in RIPA buffer with Protease and Phosphatase Inhibitor Cocktails, followed by scraping. Proteins were resolved on SDS-PAGE 4–15% and transferred to nitrocellulose membranes. Membranes were incubated in blotting solution (5% non-fat dry milk in TBS/0.1% Tween-20) at room temperature for 1 h before overnight incubation with primary antibodies. After overnight incubation at 4 °C, the blots were washed in TBS/0.1% Tween-20, followed by incubation with secondary antibodies. The blots were exposed to enhanced chemiluminescence (ECL) Western blotting substrate, and images were acquired. Protein expression was quantified by assessment of optical density of protein bands and standardised against GADPH protein expression. Primary antibodies used were anti-JNK1 + JNK2 (phospho T183 + Y185; 1:1000) antibody, anti-ERK1/2 (phospho Thr202/Tyr204; 1:1000) antibody and anti-GAPDH antibody (1:1000) and rabbit anti-ZO-1 (1:1000). Secondary antibodies used were goat anti-rabbit IgG H&L (HRP) conjugated antibody (1:10,000) and rabbit anti-mouse IgG H&L (HRP) conjugated antibody (1:10,000).

### 4.8. Cytotoxicity

To determine the integrity of the cell membrane after exposure to SCFA or HDM, the release of the cytosolic enzyme lactate dehydrogenase (LDH) was determined using the cytotoxicity detection kit^Plus^ from Roche Diagnostics (Almere, Flevoland, The Netherlands). Cytotoxic effects of exposure to the above-mentioned substances were determined by growing 16HBE cells on a collagen/fibronectin-coated 96-well plate until microscopic confluence was reached. Next, the cells were exposed to the various concentrations of SCFA or HDM applied in the experiments for 72 h. The cytotoxic effects of daily exposure to these substances for 11 days were determined by analysing the supernatant of the basolateral compartment of the inserts used in the first series of experiments (effects of SCFA on development of barrier function). Maximum release of LDH was achieved by lysing the cells with a 1% Triton X-100 solution in culture medium for 10 min at 37 °C. Thereafter, 100 µL of the supernatant was transferred four times to a 96-well plate, and 100 µL of the reaction mix of the cytotoxicity kit was added to each well. The plate was then incubated for 30 min at room temperature. Subsequently, absorbance was determined at 492 nm using a microplate reader (iMark microplate reader, Bio-Rad Laboratories, Veenendaal, Utrecht, The Netherlands), and the absorbance values of the four replicates were averaged.

### 4.9. Statistical Analysis

All data are expressed as mean ± standard error from mean (SEM). The results of the LDH assays, the TEER data of the first series of experiments, and the ELISA data were analysed using a one-way factorial analysis of variance (ANOVA), followed by Dunnett’s multiple comparison test. The results of the experiments investigating the restorative effects of SCFA on the barrier function have been analysed with a two-way ANOVA, also followed by Dunnett’s multiple comparison test. *p* values < 0.05 were considered as significant. The statistical analysis was performed using GraphPad Prism 6.14 (GraphPad Software, San Diego, CA, USA).

## Data Availability

Raw data is stored at Utrecht University. The datasets used and/or analysed during the current study are available from the corresponding author on reasonable request.

## Abbreviations

DMEM	Dulbercco’s Modified Eagle Medium
ECL	enhanced chemiluminescence
ERK1/2	extracellular signal-regulated protein kinases 1/2
FBS	fetal bovine serum
HDAC	histone deacetylases
HDM	house dust mite
JNK	c-Jun N-terminal kinases
LDH	lactate dehydrogenase
MAPK	mitogen-activated protein kinases
MEM	minimal essential medium
SCFA	Short-chain fatty acids
TEER	trans-epithelial electrical resistance
TJs	tight junctions
ZO-1	zonula occludens-1
